# 72-hour SOFA changes and risk stratification for invasive mechanical ventilation in patients with community-acquired Pneumonia

**DOI:** 10.1038/s41598-026-44586-2

**Published:** 2026-03-17

**Authors:** Genyan Liu, Jiangqin Ou, Tong Yang, Ying Huang, Banghai Feng

**Affiliations:** 1 The First Clinical Medical College of Guizhou University of TCM, Guizhou University of Traditional Chinese Medicine, No.71 Baoshan North Road, Yunyan District, Guiyang, 550001 Guizhou China; 2 Affiliated Traditional Chinese Medicine Hospital of Zunyi Medical and Pharmaceutical College (Zunyi Hospital of Traditional Chinese Medicine), No. 8, North Section of Ping’an Avenue, Honghuagang District, Zunyi, 563006 Guizhou China

**Keywords:** Community-Acquired Pneumonia, SOFA score, Invasive mechanical ventilation, Risk stratification, Dynamic assessment, $\Delta$SOFA, Diseases, Health care, Medical research, Risk factors

## Abstract

Accurate prediction of invasive mechanical ventilation (IMV) requirement in patients with community-acquired pneumonia (CAP) is a key clinical challenge. Conventional static risk stratification scores have limited value for 72-hour risk assessment, and the incremental prognostic value of dynamic organ dysfunction changes for IMV prediction remains to be systematically verified. This study aimed to evaluate the predictive value of 72-hour changes in the Sequential Organ Failure Assessment score ($\Delta$SOFA) for IMV requirement during hospitalization in CAP patients, and to develop a practical dynamic risk stratification tool designed for the 72-hour evaluation window. This was a retrospective cohort study based on the publicly available NACef database, which included 768 hospitalized CAP patients from a tertiary hospital in Colombia. After applying strict inclusion/exclusion criteria (hospital stay $\ge$72 hours, complete admission/72-hour SOFA scores and IMV outcome data), 581 patients were included in the final analysis. We constructed a dual-dimensional ABCD dynamic risk stratification framework based on admission SOFA score (<2 vs. $\ge$2) and 72-hour $\Delta$SOFA trajectory ($\le$ 0 vs. >0), stratifying patients into four subgroups: Low-Risk Stable (A), Low-Risk Deteriorating (B), High-Risk Stable (C), and High-Risk Deteriorating (D). Two logistic regression models (a static model with admission CURB-65, PSI and admission SOFA risk; a dynamic model adding $\Delta$SOFA trajectory) were developed to predict IMV requirement. The predictive performance of the models was comprehensively evaluated in terms of discrimination, calibration, reclassification improvement and clinical utility, with internal validation via 10-fold cross-validation. The four ABCD subgroups exhibited significantly different IMV rates: Group A (5.8%, 4/69), Group B (33.3%, 13/39), Group C (30.3%, 106/350), and Group D (81.3%, 100/123) ($\chi^{2}$ =136.90, $P<0.001$). Compared with Group A (reference), Group D had a markedly elevated adjusted odds ratio (aOR) for IMV (91.81, 95% CI: 28.47–375.09, $P<0.001$), followed by Group B (aOR: 12.60, 95% CI: 3.32–56.94, $P<0.001$) and Group C (aOR: 5.78, 95% CI: 2.07–20.99, $P<0.001$). The dynamic model achieved superior discriminative ability (AUC=0.850, 95% CI: 0.817–0.882) compared with the static model (AUC=0.741, 95% CI: 0.700–0.783), with a statistically significant improvement in AUC ($\Delta$ AUC=0.109,$P<0.001$) .$\Delta$SOFA worsening ($\Delta$SOFA>0) was independently associated with IMV (aOR: 13.77, 95% CI: 8.30–23.66, $P<0.001$). The dynamic model also showed better calibration (Hosmer–Lemeshow P=0.443, Brier score=0.149 vs. static model: P=0.732, Brier score=0.195), significant reclassification improvement (NRI=0.4824, IDI=0.1919, both $P<0.001$) and higher clinical utility (net benefit AUC=0.1287 vs. static model: 0.1063) across clinically relevant threshold probabilities (0–0.5). The optimal risk threshold for the dynamic model was 0.37, with a sensitivity of 65.5% and positive predictive value (PPV) of 77.2% for identifying high-IMV-risk patients. For hospitalized CAP patients with a hospital stay of $\ge$72 hours, $\Delta$SOFA within 72 hours is an independent and valuable predictor of IMV requirement in CAP patients, with significant incremental prognostic value when added to conventional static risk scores. The ABCD dynamic risk stratification framework constructed based on admission SOFA and 72-hour $\Delta$SOFA can effectively stratify CAP patients into distinct risk subgroups with clear IMV risk gradients, and the corresponding dynamic prediction model has excellent predictive performance and clinical utility. This framework provides a simple, operable dynamic risk assessment tool for the 72-hour clinical node, supplementing incremental IMV risk information for routine disease reassessment and supporting the paradigm shift in CAP risk assessment from single static admission evaluation to comprehensive “admission + dynamic trajectory” assessment.

## Introduction

Community-acquired pneumonia (CAP) remains one of the leading causes of hospitalization and mortality worldwide^1^. With global population aging and the rising prevalence of comorbidities, its disease burden continues to escalate. CAP clinical management involves sequential assessments at critical time points: initial evaluation at admission and re-evaluation shortly after hospitalization. International guidelines explicitly recommend evaluating the clinical response to initial therapy at 48–72 hours after treatment initiation^2^. Thus, how to assess patient risk and guide subsequent clinical decisions at 48–72 hours has become an important research direction in the field of CAP. At 72 hours after admission, initial treatment has been implemented and the patient’s early response has emerged, rendering this time point critical for re-evaluating disease progression and adjusting therapeutic strategies. Unlike death—an irreversible terminal event—invasive mechanical ventilation (IMV) represents a clinically actionable milestone in the course of CAP deterioration^3^. Therefore, the ability to accurately identify patients at high risk of requiring IMV at 72 hours directly informs the optimal adjustment of clinical treatment decisions.

The Pneumonia Severity Index (PSI) and CURB-65 score are currently the most widely used risk stratification tools for CAP. PSI enables comprehensive risk stratification but requires multiple clinical parameters, whereas CURB-65 is concise and efficient, making it more suitable for emergency settings. Extensive research has confirmed the stable efficacy of these two tools in predicting short-term mortality in CAP patients, and they play an irreplaceable role in initial admission evaluation^4^.

To address the need for 72-hour risk evaluation and IMV prediction, numerous advances have been made in recent years. Some studies have constructed predictive models using machine learning algorithms, demonstrating promising accuracy in predicting IMV requirements. Another study developed a LightGBM model incorporating 23 variables to predict mortality in ICU patients with severe CAP, achieving an AUC of 0.856 in external validation^5^.Przybilla et al. developed a continuous-time Markov model based on the Sequential Organ Failure Assessment (SOFA) score and found that the disease course of CAP follows a Markov process, highlighting the necessity of daily clinical monitoring and repeated patient evaluation^6^.These studies provide valuable insights into the dynamic risk assessment of CAP, although their complexity—requiring extensive variables or computationally intensive algorithms—limits routine application at the 72-hour reassessment window in general ward settings.

However, several critical gaps remain in the current research landscape. First, studies focusing on the specific outcome of IMV requirement are relatively scarce. The aforementioned machine learning models predominantly target mortality or composite endpoints as their prediction outcomes. Second, the applicability of existing risk stratification tools at the 72-hour time point requires further validation. Although PSI and CURB-65 can be used for dynamic monitoring, most of their components are admission characteristics or single-time-point measurements, and their value in reflecting short-term disease trends remains unconfirmed^7,8^. Third, research on dynamic changes in the SOFA score ($\Delta$SOFA) in the field of CAP remains limited^9^. Although $\Delta$SOFA has been established as a more sensitive prognostic marker than a single static SOFA score in critical illnesses such as sepsis, its specific value in predicting IMV requirements in CAP patients has not been systematically evaluated^10^. Fourth, the complexity of existing predictive models limits their routine clinical application. Machine learning models require extensive variable input, and Markov models demand high data quality, which restricts their feasibility in general ward settings.

Based on these research gaps, the present study investigates the predictive value of $\Delta$SOFA within 72 hours after admission for IMV requirements in CAP patients and evaluates its incremental prognostic value when added to conventional risk stratification tools. To address the critical need for simple, dynamic assessment at the 72-hour window, we introduce a practical ABCD stratification framework by integrating admission SOFA with its 72-hour trajectory ($\Delta$SOFA). This framework translates the dynamic response of organ function into an actionable clinical tool for early IMV risk stratification—an approach that remains underutilized in the routine management of hospitalized CAP patients. The findings are expected to provide clinicians with quantifiable evidence for decision-making at the 72-hour time point (e.g., whether to escalate monitoring or prepare for respiratory support), thereby improving patient outcomes and optimizing medical resource allocation.

## Methods

**Study design and data source**

This was a retrospective cohort study. Data were derived from the NACef (Community-Acquired Pneumonia, Endotypes, and Phenotypes) database, a prospective observational cohort conducted at the Clinica Universidad de La Sabana (Chía, Colombia) between January 2020 and July 2022^11^. The dataset is publicly available on the PhysioNet platform (DOI: 10.13026/4y3t-pq44) under the PhysioNet Restricted Health Data License 1.5.0^12^.

The database comprises comprehensive de-identified clinical information from 768 patients diagnosed with CAP, including demographic characteristics, clinical outcomes, admission characteristics, in-hospital follow-up, and post-discharge data. Access to this de-identified dataset requires completion of the PhysioNet credentialing process, including signing a Data Use Agreement and CITI training on human subjects research, in compliance with the HIPAA Safe Harbor Provision.

**Ethical approval and consent to participate**

The NACef study was approved by the Ethics Committee for Research Quality and Ethical Integrity of Clinica Universidad de La Sabana (Approval No. 021, February 4, 2020)^11^. Written informed consent was obtained from all participants or their legally authorized representatives. All data were de-identified in accordance with HIPAA standards, including removal of direct identifiers, age top-coding (>90 years aggregated), date shifting to relative days, and outlier masking. As a secondary analysis of publicly available anonymized data, this study was exempt from additional ethical review. All procedures were performed in accordance with the Declaration of Helsinki.

**Study population and inclusion/exclusion criteria**

The initial cohort of 768 patients was derived from the NACef database, with the original enrollment criteria as follows^11^:

**Original database inclusion criteria**

1. Aged $$> 18$$ years;
2. Met the pneumonia diagnostic criteria of the American Thoracic Society/Infectious Diseases Society of America (ATS/IDSA);
3. Admitted to hospital within 24 hours of symptom onset;
4. Written informed consent provided by the patient or legal representative.

**Original database exclusion criteria**

1. Unable to provide informed consent;
2. With active tuberculosis;
3. Uncertain diagnosis or higher likelihood of alternative diagnoses;
4. Severe missing clinical data precluding analysis (as defined in the original protocol).

Based on the original cohort, we further screened patients who met the requirements for dynamic assessment of invasive mechanical ventilation (IMV) outcomes at 72 hours, with the specific criteria as follows:

**Inclusion criteria for this study**

1. Hospital stay $\ge 72$ hours;
2. Community-acquired pneumonia (CAP) as the primary diagnosis at admission.

**Exclusion criteria for this study**

1. Missing admission or 72-hour Sequential Organ Failure Assessment (SOFA) scores;
2. Missing data on IMV outcomes.

Patients who required IMV before 72 hours were included in the analysis, provided they had complete SOFA data at both time points; for these patients, the 72-hour SOFA score was calculated using post-intubation clinical data.

**Handling of missing data**

For predictor variables with minimal missingness, single imputation was performed to preserve the analytic sample: missing admission PSI scores ($n=4$) were imputed with the median, missing age ($n=7$) with the median, and missing sex ($n=1$) with the mode.For BMI, which had more missing data, multiple imputation by chained equations (MICE) with 5 imputations was performed. The imputation model included age, sex, and key severity scores (CURB-65, PSI, admission SOFA) to preserve the relationships among these variables. Convergence was assessed by inspecting trace plots of imputed values across iterations. The pooled estimates from the five imputed datasets were used for descriptive analyses (e.g., Table 1). Of note, BMI was not included as a predictor in any of the multivariable regression models; therefore, the handling of missing BMI data does not affect the primary predictive analyses.

**Fig. 1:**
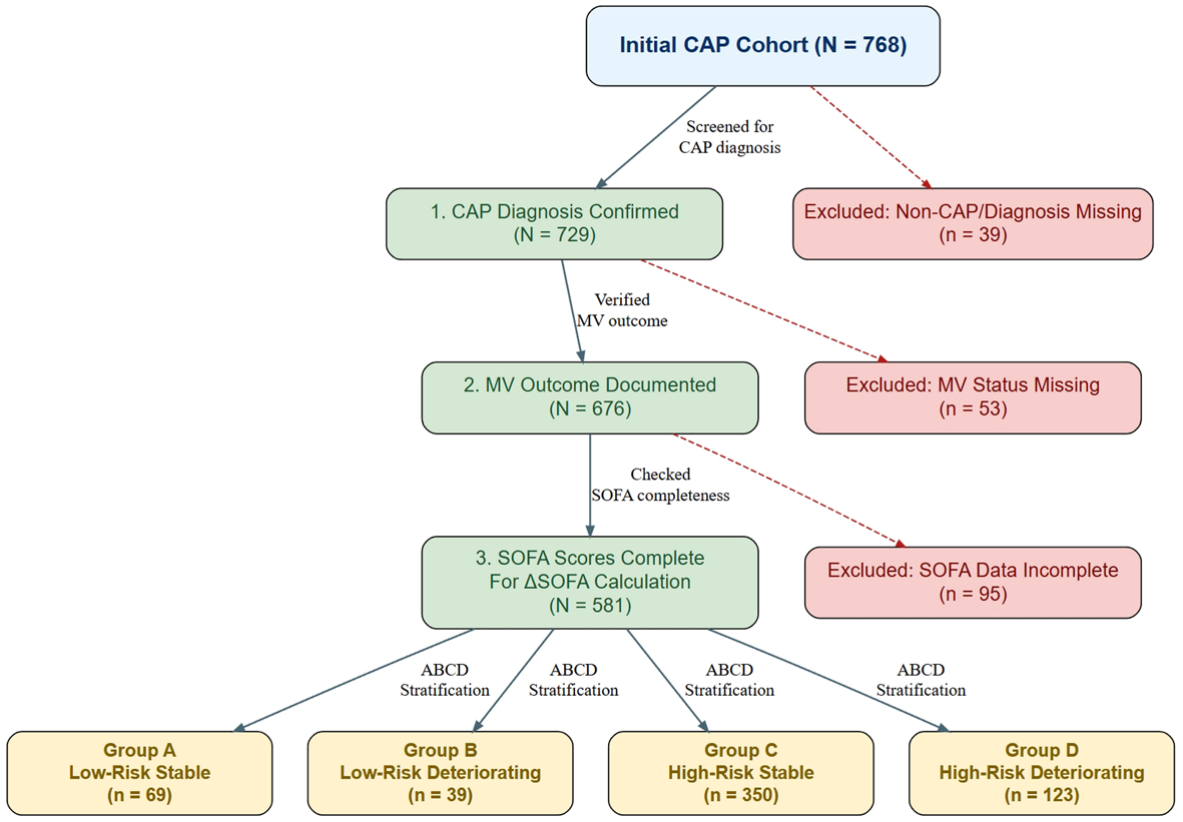
Study cohort enrollment and stratification flowchart. A total of 768 patients were initially screened; 187 patients were excluded based on diagnosis, missing outcome, and incomplete SOFA data. The final analytic sample comprised 581 patients, who were stratified into four risk-deterioration groups as depicted.

Based on the above criteria, a total of 187 patients were excluded from the initial cohort of 768 patients: 53 due to missing IMV outcome data, 95 due to incomplete SOFA scores (either at admission or at 72 hours, including those who died or were discharged within 72 hours), and 39 because CAP was not the primary diagnosis. The remaining 581 patients constituted the final analysis cohort. The patient selection flowchart is presented in Fig. 1.

**Variable definitions**

**Primary exposure variables**

- **Admission SOFA score**: Assessed within 24 hours of hospital admission in accordance with international consensus criteria, encompassing six organ systems (respiratory, coagulation, liver, cardiovascular, renal, and neurological). The total score ranges from 0 to 24, with higher scores indicating more severe organ dysfunction.
- **72-hour SOFA score**: Reassessed at 72 ± 2 hours after admission to capture dynamic changes in organ function following initial clinical intervention.
- $\Delta$**SOFA**: Defined as the difference between the 72-hour SOFA score and the admission SOFA score (variable name: $\Delta$SOFA). A positive $\Delta$SOFA ($\Delta$SOFA > 0) indicates organ function deterioration, while a non-positive $\Delta$SOFA ($\Delta$SOFA $\le$ 0) indicates stability or improvement^13^.

**Patient group stratification**

Based on the admission SOFA score (for risk stratification) and $\Delta$SOFA (for dynamic evolution), eligible patients were divided into four mutually exclusive groups:

1. **Group A (Low-Risk Stable)**: admission SOFA < 2 and $\Delta$SOFA $\le$ 0;
2. **Group B (Low-Risk Deteriorating)**: admission SOFA < 2 and $\Delta$SOFA > 0;
3. **Group C (High-Risk Stable)**: admission SOFA $\ge$ 2 and $\Delta$SOFA $\le$ 0;
4. **Group D (High-Risk Deteriorating)**: admission SOFA $\ge$ 2 and $\Delta$SOFA > 0.

**Outcome variables**

The primary outcome of this study was the requirement for **invasive mechanical ventilation (IMV)** during hospitalization. In the NACef database, ventilatory support was recorded under the variable *ventilation*, encompassing invasive ventilation, non-invasive ventilation, and high-flow nasal cannula. According to the source publication, 41.4% (318/768) of patients received any form of ventilatory support, of whom 81.9% underwent exclusively invasive ventilation^14^. For the present analysis, we therefore restricted the outcome to **invasive ventilation only**, based on the specific ventilation type coded in the database. Within the final analytic cohort of 581 patients, the overall IMV rate was **38.4% (223/581)**.

Although the decision to initiate IMV was not governed by a rigid, study-specific protocol due to the retrospective design, its clinical validity is supported by the rigorous framework of the original NACef cohort. As described in the primary study^14^, all CAP diagnoses and subsequent management decisions were made by a multidisciplinary team—including infectious disease physicians, internal medicine specialists, and intensivists—in accordance with international guidelines. This process minimized diagnostic and therapeutic heterogeneity and ensured that the recorded IMV events reflect expert consensus in real-world clinical practice.

**Predictor variables for modeling**

To predict IMV occurrence, two logistic regression models were constructed with the following predictors:

- **Static model**: Included the admission CURB-65 score (continuous), admission Pneumonia Severity Index (PSI score, continuous), and dichotomized admission SOFA risk (*sofa_risk*: 0 = < 2 points, 1 = $\ge$ 2 points).
- **Dynamic model**: Included all predictors of the static model plus a dichotomized $\Delta$SOFA trajectory (*delta_risk*: 0 = $\Delta$SOFA $\le$ 0, 1 = $\Delta$SOFA > 0).

Both the admission CURB-65 score and admission PSI score were treated as continuous variables, whereas *sofa_risk* and *delta_risk* were categorized as binary categorical variables, with the low-risk group (*sofa_risk* = 0) and the stable group (*delta_risk* = 0) set as the reference categories. No additional variable selection was performed; all predictors were prespecified based on established clinical associations with CAP severity and IMV risk.

**Statistical analysis**

***Sample Size and Power Consideration*** With a final analytic cohort of 581 patients and 223 IMV events (event rate 38.4%), the study had adequate statistical power for the planned analyses. Based on a two-sided significance level of $\alpha = 0.05$, this sample size provides $>95\%$ power to detect an odds ratio of 1.5 for a one-standard-deviation increase in a continuous predictor, assuming a moderate correlation ($R^2 = 0.3$) with other covariates. Furthermore, the events per predictor (EPV) ratio was 55.8 (223 events/4 predictors), substantially exceeding the recommended minimum of 10 EPV, indicating a low risk of model overfitting^14^.

***Data Source and Outcome Ascertainment*** All predictors and the outcome (IMV) were objectively derived from electronic medical records with standardized protocols in the original NACef database. Therefore, no additional blinding or inter-rater reliability assessment was performed.

***Software and Statistical Threshold*** All statistical analyses were performed using R software (version 4.5.1; R Foundation for Statistical Computing, Vienna, Austria)^15^ with the following packages: pROC (version 1.18.0)^16^, rms (version 6.5-0.5.5.5)^17^, ggplot2 (version 3.4.2)^18^, and rmda (version 1.6)^19^. A two-tailed *P*-value $<0.05$ was considered statistically significant. All performance estimates were accompanied by 95% confidence intervals (CIs) derived from bootstrap resampling (1,000 replicates) where applicable.

**Descriptive statistics**

- **Continuous variables:** Due to non-normal distribution (Shapiro–Wilk test, all $P<0.05$), data were presented as median (interquartile range, IQR). Comparisons across the four ABCD risk groups were conducted using the Kruskal–Wallis *H* test, with pairwise comparisons performed where appropriate.
- **Categorical variables:** Data were expressed as frequencies (percentages) and compared using the Pearson $\chi ^{2}$ test; Fisher’s exact test was applied when the expected count of any cell was $<5$. Between-group differences in the primary outcome (IMV rate) were evaluated using the $\chi ^{2}$ test, with pairwise comparisons adjusted for multiple testing (Bonferroni correction).

**Prediction Model Development** Two binary logistic regression models were prespecified as defined above. No formal sample size calculation was performed *a priori*; however, the number of IMV events per candidate predictor in the dynamic model (4 predictors) was approximately 55.8, far exceeding the recommended minimum of 10^14^.

**Model Evaluation** The predictive performance of the static and dynamic models was assessed across three core dimensions: discrimination, calibration, and clinical utility, with additional analyses for reclassification improvement and threshold optimization.

**Discrimination**

- Receiver operating characteristic (ROC) curves were plotted, and the area under the curve (AUC) with 95% CI was calculated for each model using the pROC package.
- The **DeLong test** was used to compare AUC values between the two models.
- Adjusted odds ratios (aOR) with 95% CI for $\Delta$SOFA worsening (vs. stable/improved) were extracted directly from the dynamic model’s logistic regression coefficients.
- Sensitivity, specificity, and Youden index were calculated across all probability thresholds to identify the optimal cut-off for risk stratification (based on maximum Youden index).

**Calibration**

- **Calibration curves** were constructed by stratifying predicted probabilities into deciles (10 equal groups) and plotting the mean predicted probability against the observed event proportion within each group.
- 95% CIs for calibration curves were estimated using **bootstrap resampling** (1,000 replicates) via the rms package and visualized with ggplot2.
- The **Hosmer–Lemeshow (HL) goodness-of-fit test** (with 10 groups) was performed to quantify agreement between predicted and observed outcomes, with $P>0.05$ indicating no significant calibration bias.
- The **Brier score** was computed as a global measure of prediction accuracy (range: 0–1; lower values indicate better overall performance).

**Internal validation and model comparison**

- Stratified 10-fold cross-validation with 5 repetitions was performed to derive optimism-corrected AUC and assess internal validity. The distribution of key characteristics across folds was balanced (Supplementary Table S3), confirming the robustness of the validation procedure.
- The **Bayesian information criterion (BIC)** was calculated for both models to balance goodness-of-fit and model complexity.

**Reclassification improvement**

- **Net reclassification improvement (NRI)** and **integrated discrimination improvement (IDI)** were calculated to quantify the incremental value of adding $\Delta$SOFA trajectory to the static model.
- Event-specific and non-event-specific components of NRI and IDI were reported, with 95% CIs obtained via bootstrap (1,000 replicates).

**Clinical utility**

- **Decision curve analysis (DCA)** was performed using the rmda package to evaluate the net benefit of each model across a range of clinically relevant threshold probabilities (0–0.5), compared to the “treat all” and “treat none” strategies.
- Net benefit AUC was calculated to summarize the overall clinical utility of each model.
- **Risk threshold analysis** was conducted to determine the optimal probability threshold (based on maximum Youden index) for identifying high-risk patients, with corresponding sensitivity, positive predictive value (PPV), and high-risk proportion reported for both models.

**Model Presentation** The final dynamic model included the following predictors: admission CURB-65 score (continuous), admission PSI score (continuous), admission SOFA risk (dichotomized as $<2$ vs $\ge 2$), and 72-hour $\Delta$SOFA trajectory (dichotomized as $\le 0$ vs $>0$). The regression coefficients, standard errors, adjusted odds ratios with 95% confidence intervals, and corresponding *P*-values were calculated. **Code and Data Availability** The R code for data preprocessing, model development, and performance evaluation is publicly available on OSF and archived with the DOI: 10.17605/OSF.IO/HNES3 (https://osf.io/hnes3).The NACef source dataset can be accessed via PhysioNet (DOI: 10.13026/4y3t-pq44) under the PhysioNet Restricted Health Data License 1.5.0. The cleaned analysis dataset, derived from the original data with no additional modifications, is available from the corresponding author upon reasonable request, subject to the same license terms.

## Results

### Baseline characteristics of the study cohort

**Table 1:** Baseline characteristics of included and excluded patients.

Characteristic	Excluded (*n*=187)	Included (*n*=581)	*p*-value	Test
Age, median [IQR]	65 [54, 74]	62 [51, 72]	0.218	Wilcoxon
BMI, median [IQR]	27.76 [24.22, 30.36]	27.13 [24.60, 30.11]	0.798	Wilcoxon
Admission SOFA, median [IQR]	2.00 [2.00, 3.00]	3.00 [2.00, 4.00]	0.015	Wilcoxon
CURB-65, median [IQR]	1.00 [0.00, 2.00]	1.00 [0.00, 2.00]	0.056	Wilcoxon
PSI, median [IQR]	74.00 [57.00, 92.75]	76.00 [57.00, 101.00]	0.239	Wilcoxon
Male, *n* (%)	113 (60.4)	369 (63.6)	0.485	$\chi ^2$
COPD, *n* (%)	19 (10.2)	63 (10.8)	0.899	$\chi ^2$

Note: Continuous variables are presented as median [interquartile range]; categorical variables are presented as *n* (%). Wilcoxon = Wilcoxon rank-sum test; $\chi ^2$ = Chi-square test.

**Included vs. Excluded Patients** The baseline characteristics of the 581 included patients and 187 excluded patients are summarized in Table 1. No significant differences were observed in age, body mass index (BMI), gender, or chronic obstructive pulmonary disease (COPD) prevalence between the two groups (all $P>0.05$), indicating that the excluded patients did not systematically differ from the included cohort. However, included patients had a significantly higher median admission SOFA score (3.00 [IQR: 2.00, 4.00] vs. 2.00 [IQR: 2.00, 3.00]; $P=0.015$), reflecting the exclusion of patients with incomplete SOFA data or hospital stays $<72$ hours.

**ABCD Risk-Deterioration Groups** The 581 patients were stratified into four mutually exclusive groups based on admission SOFA score and $\Delta$SOFA trajectory (Table 2):

**Table 2:** Baseline characteristics of the study participants by ABCD risk groups.

Characteristic	A: Low-Risk Stable	B: Low-Risk Deteriorating	C: High-Risk Stable	D: High-Risk Deteriorating	*p*-value
*n*	69	39	350	123	
Age years, median [IQR]	55 [41, 66]	57 [45, 66]	62 [51, 72]	58 [49, 69]	0.008
Male, *n* (%)	27 (39.1)	10 (25.6)	135 (38.6)	39 (31.7)	0.251
BMI, median [IQR]	26.67 [23.05, 29.7]	26.99 [23.63, 28.89]	26.67 [24.13, 29.86]	27.76 [25.76, 30.64]	0.044
CURB-65 score, median [IQR]	1 [0, 2]	1 [0, 1]	1 [1, 2]	1 [1, 2]	$<0.001$
PSI score, median [IQR]	60 [45, 83]	53 [42.5, 71.5]	80 [60.25, 103]	82 [67.5, 111]	$<0.001$
Admission SOFA score, median [IQR]	1 [0, 1]	0 [0, 1]	3 [2, 5]	3 [2, 4.5]	$<0.001$

Note: Demographic and clinical characteristics of the study population, stratified by the ABCD risk groups. Data are presented as median [interquartile range] for continuous variables and *n* (%) for categorical variables.

- **Group A (Low-Risk Stable)**: 69 patients (11.9%) with admission SOFA $<2$ and $\Delta$SOFA $\le 0$;
- **Group B (Low-Risk Deteriorating)**: 39 patients (6.7%) with admission SOFA $<2$ and $\Delta$SOFA $>0$;
- **Group C (High-Risk Stable)**: 350 patients (60.2%) with admission SOFA $\ge 2$ and $\Delta$SOFA $\le 0$;
- **Group D (High-Risk Deteriorating)**: 123 patients (21.2%) with admission SOFA $\ge 2$ and $\Delta$SOFA $>0$.

Significant between-group differences were observed for age ($P=0.008$), BMI ($P=0.044$), CURB-65 score ($P<0.001$), PSI score ($P<0.001$), and admission SOFA score ($P<0.001$), while gender distribution was comparable across groups ($P=0.251$). Patients in Group D were older and had higher severity scores, consistent with their high-risk deteriorating phenotype.

### Association between ABCD risk groups and invasive mechanical ventilation

**Fig. 2:**
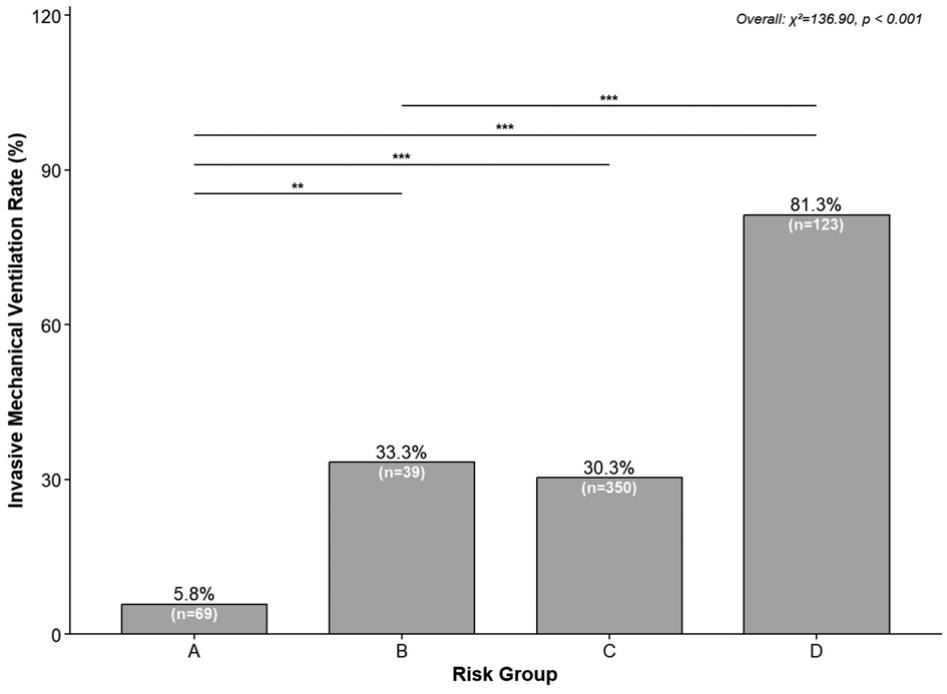
Rate of invasive mechanical ventilation (IMV) across ABCD risk-deterioration groups. Significant between-group differences were detected by chi-square test (overall $\chi ^2_{3} = 136.90$, $P < 0.001$). Triple asterisks (***) denote $P < 0.001$ in pairwise comparisons (Bonferroni-corrected).

The primary outcome of invasive mechanical ventilation (IMV) occurred in 38.4% (223/581) of the final cohort. IMV rates varied dramatically across the four risk groups (Fig. 2):

- **Group A**: 5.8% (4/69);
- **Group B**: 33.3% (13/39);
- **Group C**: 30.3% (106/350);
- **Group D**: 81.3% (100/123).

Overall between-group differences were highly significant ($\chi ^2 = 136.90$, $P<0.001$). Pairwise comparisons (Bonferroni-corrected) confirmed that Group D had the highest IMV rate (all $P<0.001$ vs. Groups A/B/C), while Group A had the lowest (all $P<0.01$ vs. Groups B/C/D).

**Fig. 3:**
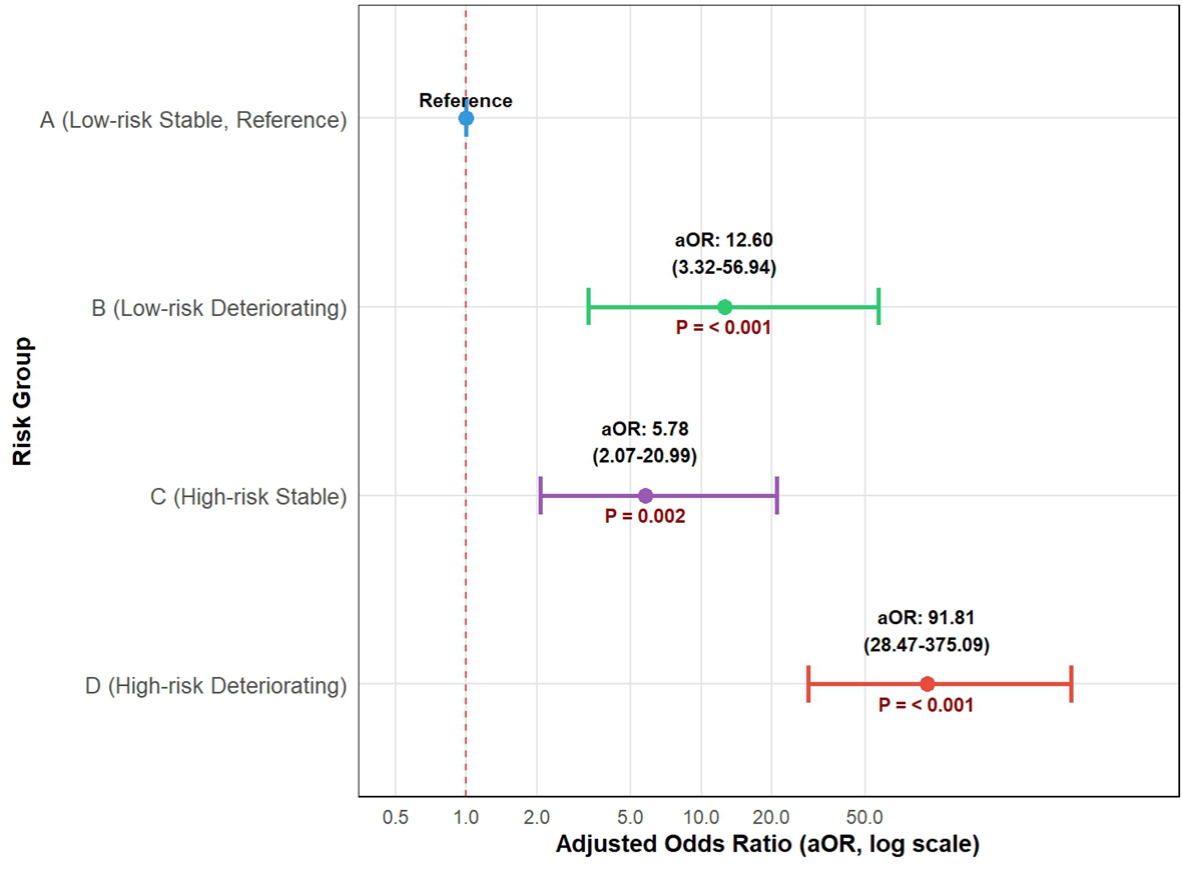
Forest plot of adjusted odds ratios (aOR) across risk-deterioration groups. Group A served as the reference group. Adjusted ORs, 95 % confidence intervals and P-values were estimated for the primary outcome. The x-axis was displayed on a logarithmic scale for better visualization. Confidence interval widths reflect subgroup sample sizes and the magnitude of effect estimates.

In adjusted logistic regression (Fig. 3), Group D was associated with a 91.81-fold increased odds of IMV (adjusted odds ratio [aOR]: 91.81, 95% CI: 28.47–375.09; $P<0.001$) compared to Group A (reference). Group B (aOR: 12.60, 95% CI: 3.32–56.94; $P<0.001$) and Group C (aOR: 5.78, 95% CI: 2.07–20.99; $P<0.001$) also demonstrated significantly elevated odds of IMV.

### Prediction model performance

**Fig. 4:**
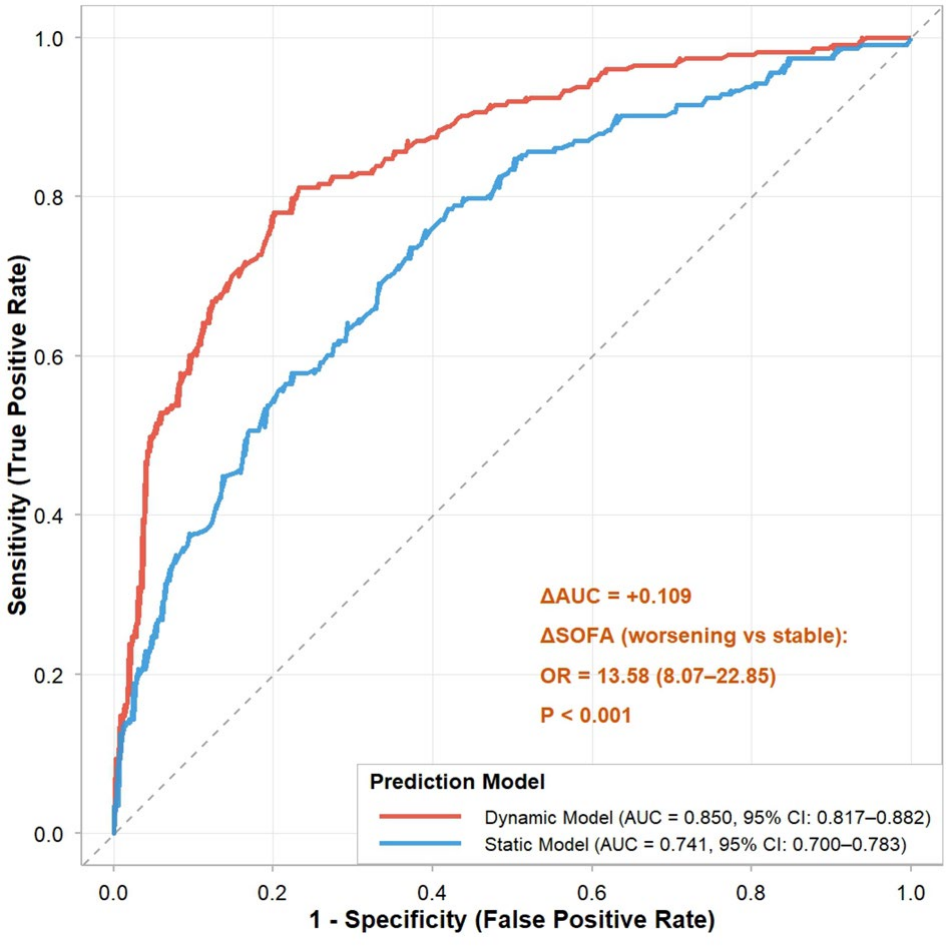
Comparison of ROC curves between the dynamic and static prediction models. ROC curves for the dynamic (red) and static (blue) models, with corresponding AUC, 95% CI, CV-AUC and BIC values shown. The dashed diagonal line denotes the reference line.

**Discrimination**

The discriminative ability of the static and dynamic models for predicting IMV is shown in Fig. 4. The static model (admission CURB-65 + PSI + *sofa_risk*) achieved an area under the curve (AUC) of 0.741 (95% CI: 0.700–0.783). The dynamic model, which additionally included *delta_risk*, demonstrated superior discrimination with an AUC of 0.850 (95% CI: 0.817–0.882). The DeLong test confirmed a statistically significant improvement ($\Delta$AUC = 0.109, $P < 0.001$). In the dynamic model (Table 3), $\Delta$SOFA worsening (*delta_risk* = 1) was independently associated with a 13.77-fold increased odds of IMV (OR: 13.77, 95% CI: 8.30–23.66; $P < 0.001$), along with CURB-65 (OR 1.89, 95% CI 1.48–2.43), PSI (OR 1.01, 95% CI 1.00–1.02), and admission SOFA $\ge 2$ (OR 4.91, 95% CI 2.56–9.97).

**Table 3:** Multivariable logistic regression model for predicting invasive mechanical ventilation (dynamic model).

Variable	Coefficient ($\beta$)	Standard Error	Odds Ratio (OR)	95% CI	*P*-value
Intercept	$-4.425$	0.427	0.01	0.00–0.03	$<0.001$
CURB-65 (per 1-point increase)	0.636	0.127	1.89	1.48–2.43	$<0.001$
PSI (per 1-point increase)	0.011	0.004	1.01	1.00–1.02	0.002
Admission SOFA $\ge 2$ (vs $<2$)	1.592	0.346	4.91	2.56–9.97	$<0.001$
$\Delta$SOFA $>0$ (vs $\le 0$)	2.622	0.267	13.77	8.30–23.66	$<0.001$

*Abbreviations*: OR, odds ratio; CI, confidence interval; SOFA, Sequential Organ Failure Assessment. The model was adjusted for CURB-65 score, PSI score, admission SOFA risk ($\ge 2$ vs $<2$), and 72-h $\Delta$SOFA ($>0$ vs $\le 0$).

**Calibration**

**Fig. 5:**
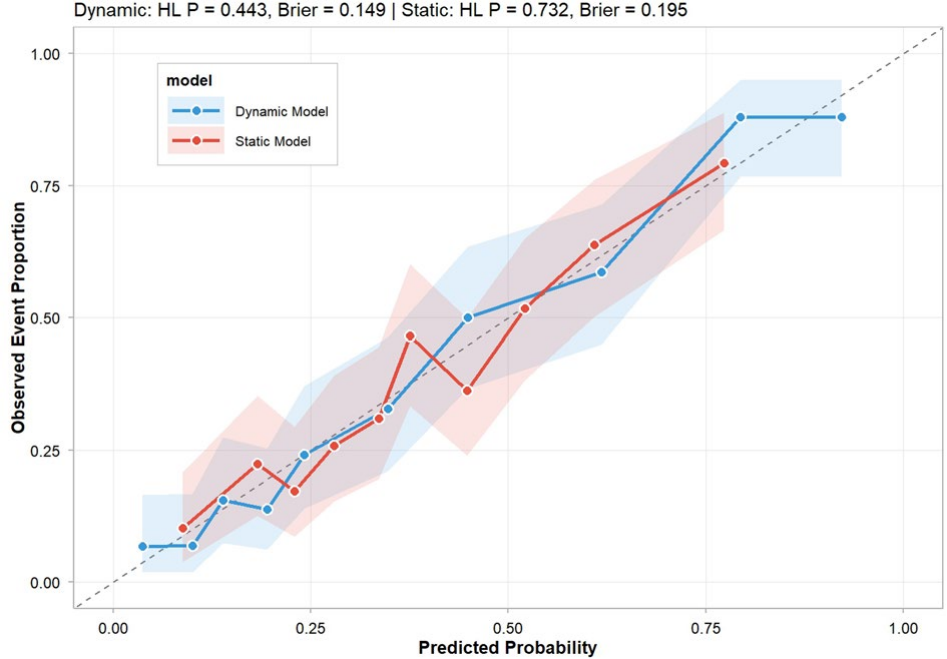
Calibration curves of the dynamic and static prediction models. The blue and orange lines represent the dynamic model and static model, respectively. Shaded areas denote 95% confidence intervals. Metrics: dynamic model (Hosmer-Lemeshow test P=0.443, Brier score=0.149); static model (P=0.732, Brier score=0.195).

Calibration curves (Fig. 5) revealed excellent agreement between predicted probabilities and observed IMV rates for both models, with curves closely following the ideal $45^\circ$ reference line across all deciles of predicted risk. Quantitative metrics confirmed this:

- **Hosmer–Lemeshow test**: Dynamic model ($P=0.443$), Static model ($P=0.732$); both $P>0.05$, indicating no significant calibration bias.
- **Brier score**: Dynamic model = 0.149; Static model = 0.195, confirming superior overall prediction accuracy for the dynamic model.

**Reclassification improvement**

**Fig. 6:**
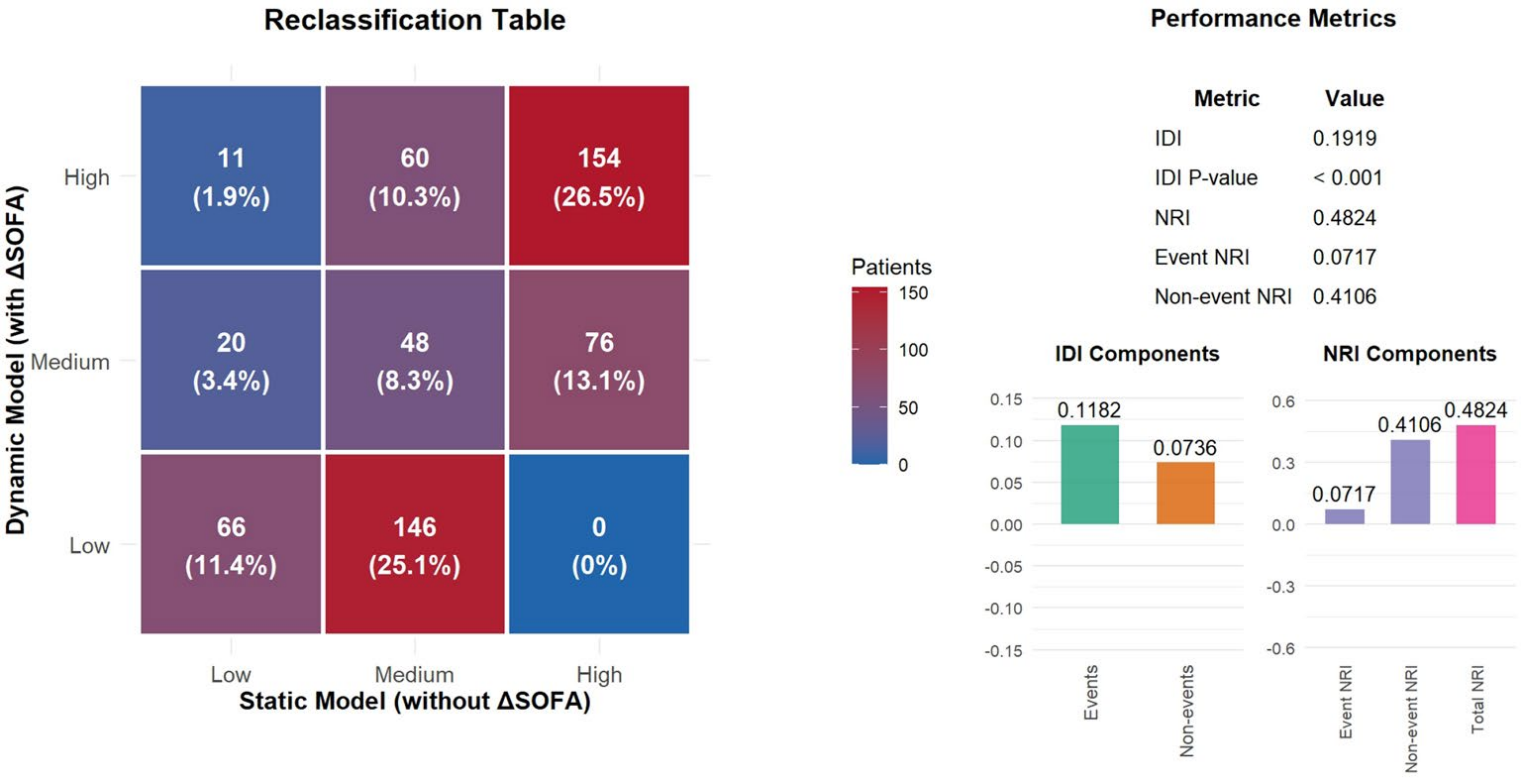
Risk reclassification and discriminatory improvement metrics between the dynamic (with $\Delta$ SOFA) and static (without $\Delta$ SOFA) models. The left panel shows the risk stratification reclassification table, and the right panels present the integrated discrimination improvement (IDI), net reclassification improvement (NRI) and their corresponding components.

Addition of $\Delta$SOFA trajectory to the static model yielded substantial reclassification improvement (Fig. 6). The integrated discrimination improvement (IDI) was 0.1919 ($P<0.001$), with event-specific IDI = 0.1182 and non-event-specific IDI = 0.0736. The net reclassification improvement (NRI) was 0.4824 ($P<0.001$), driven primarily by non-event NRI (0.4106) and supplemented by event NRI (0.0717).

**Clinical utility and Threshold analysis**

**Fig. 7:**
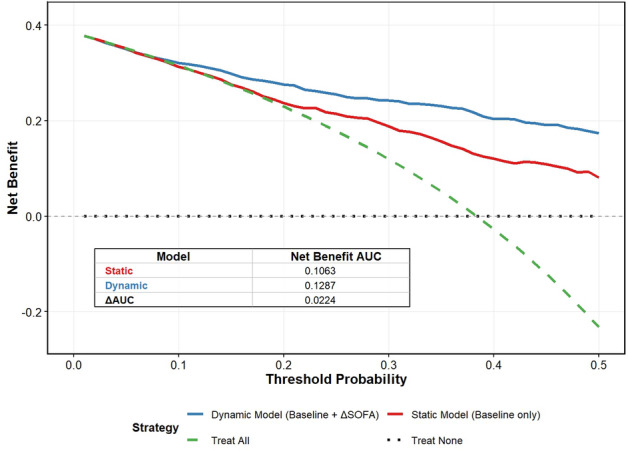
Decision curve analysis (DCA) of the dynamic and static prediction models. DCA was performed to evaluate the clinical net benefit across threshold probabilities. The blue and red curves represent the dynamic model (with $\Delta$SOFA) and static model (without $\Delta$SOFA), respectively. The green and dashed black lines denote the reference curves of treat all and treat none. Net benefit AUC of each model is also presented.

Decision curve analysis (DCA, Fig. 7) evaluated the net benefit of both models across clinically relevant threshold probabilities (0–0.5). The dynamic model consistently outperformed the static model, with higher net benefit at every threshold, and both models outperformed the “treat all” and “treat none” strategies across the entire range. Net benefit AUC was 0.1287 for the dynamic model and 0.1063 for the static model ($\Delta$AUC = 0.0224), confirming superior overall clinical utility.

**Fig. 8:**
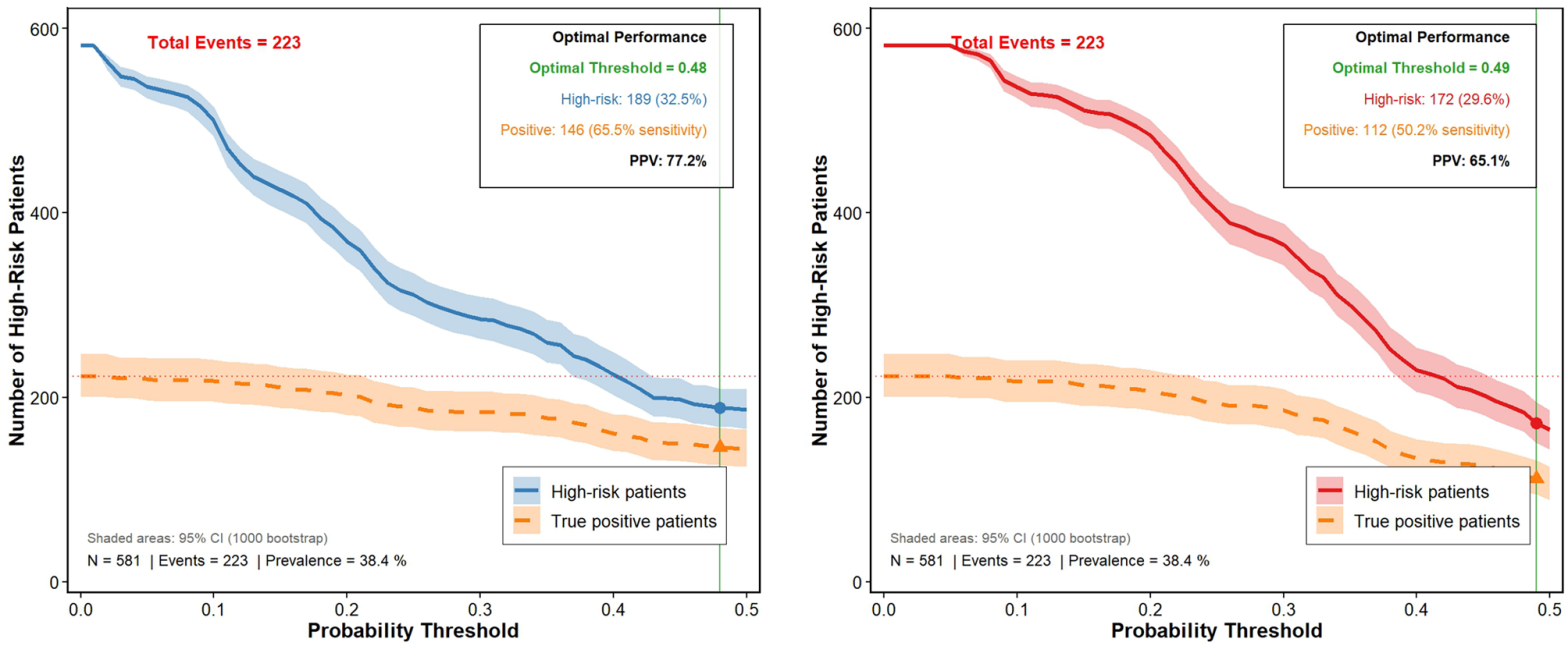
Number of high-risk and true-positive patients across probability thresholds for the two models. The left and right panels present the dynamic model (with $\Delta$SOFA) and static model (without $\Delta$SOFA), respectively. Shaded areas represent 95% confidence intervals. The optimal probability threshold, total events, and corresponding predictive indicators are annotated for each model.

Risk threshold analysis (Fig. 8) identified optimal probability cut-offs for risk stratification based on maximum Youden index:

- **Dynamic model**: Optimal threshold = 0.37, identifying 189 high-risk patients (32.5% of the cohort) with a sensitivity of 65.5% and positive predictive value (PPV) of 77.2%;
- **Static model**: Optimal threshold = 0.34, identifying 172 high-risk patients (29.6% of the cohort) with a sensitivity of 50.2% and PPV of 65.1%.

The dynamic model’s higher sensitivity and PPV at a slightly higher threshold confirm its ability to more accurately identify high-risk patients while minimizing false positives.

### Supplementary analyses

Additional analyses are presented in the Supplementary Material to support the robustness of the primary findings.

- **Figure S1** displays the distribution of the Pneumonia Severity Index (PSI) score across the four ABCD risk groups. As expected, Groups C and D (high admission SOFA) had significantly higher PSI scores compared to Groups A and B (Kruskal–Wallis test, $P<0.001$), confirming that the ABCD stratification aligns with established severity measures while incorporating dynamic trajectory information. The distribution of CURB-65 scores showed a similar pattern (as shown in Figure S1b).
- **Figure S2** illustrates the performance metrics (sensitivity, specificity, and Youden index) across all probability thresholds for both the static and dynamic models.The key observation is that the dynamic model (left panel) maintains higher sensitivity and Youden index across the majority of clinically relevant thresholds (approximately 0.2–0.5) compared to the static model (right panel). This indicates that, regardless of the chosen risk cut-off, the dynamic model achieves a better balance between true-positive and false-positive classifications. The vertical dashed lines mark the optimal thresholds (0.370 for the dynamic model, 0.340 for the static model) based on the maximum Youden index, which correspond to the values reported in the main text.
- **Table S2** presents subgroup analyses evaluating the consistency of the ABCD framework across different populations. The framework maintained a similar risk gradient across sex and age subgroups (all *P* for interaction $>0.05$). A significant interaction was observed for COPD status ($P=0.0395$), which may be influenced by the small sample sizes in COPD subgroups (e.g., only 2 patients in Groups A and B with COPD), and should be interpreted with caution.

These supplementary analyses consistently reinforce the primary conclusions: the ABCD framework captures meaningful risk stratification beyond static scores, and the dynamic model offers superior performance across the full spectrum of decision thresholds.

## Discussion

### Summary and interpretation of key findings

This retrospective cohort study was conducted in a single-center cohort of hospitalized patients with community-acquired pneumonia (CAP). We developed and validated a dynamic risk stratification framework based on the Sequential Organ Failure Assessment (SOFA) score within 24 hours of admission and the 72-hour SOFA change value ($\Delta$SOFA), and constructed static and dynamic prediction models to quantitatively evaluate the incremental predictive value of $\Delta$SOFA for invasive mechanical ventilation (IMV). The principal findings of this study are summarized as follows:

First, the novel ABCD dynamic stratification method proposed in this study, with admission organ function status and 72-hour early evolution trajectory as dual dimensions, stratified CAP patients into four subgroups with distinct risk characteristics and significantly different IMV rates, and the risk gradient of each subgroup had clear clinical identifiability: Group A (low-risk stable) 5.8%, Group B (low-risk deteriorating) 33.3%, Group C (high-risk stable) 30.3%, and Group D (high-risk deteriorating) 81.3% ($P<0.001$). These results confirmed that risk assessment relying solely on static admission indicators has limitations, and combining the dynamic changes of organ function at 72 hours can achieve more clinically meaningful IMV risk stratification; the dynamic model quantitatively verified the incremental value of $\Delta$SOFA and provided statistical support for the ABCD stratification framework.

Second, each of the four ABCD subgroups has unique clinical connotations and prognostic significance, which are organic components of the dual-dimensional stratification framework and jointly constitute a complete 72-hour dynamic risk map for CAP patients:

- **Group A (Low-risk stable: admission SOFA** $< 2$ **and** $\Delta$**SOFA** $\le 0$): Accounting for 11.9% of the study cohort, this group is the core low-risk population among CAP patients. Patients in this group had no obvious admission organ dysfunction and responded well to initial treatment within 72 hours, with organ function remaining stable or improved, and the IMV rate was only 5.8%. This indicates a good clinical prognosis, and they are low-risk populations that do not require excessive monitoring among CAP patients. This subgroup also provides a low-risk reference benchmark for the dynamic stratification framework.
- **Group B (Low-risk deteriorating: admission SOFA** $< 2$ **and** $\Delta$**SOFA** $> 0$): Accounting for 6.7% of the study cohort, this group is an early warning subgroup of initial mild disease with early progression. Patients in this group had no obvious organ dysfunction at admission but developed organ function deterioration within 72 hours, with the IMV rate surging to 33.3%. This suggests that even for CAP patients with initial mild disease, the early deterioration trajectory of organ function is an important early warning signal for IMV, and the identification of this subgroup fills the gap of insufficient recognition of “mild disease progression” by static scores.
- **Group C (High-risk stable: admission SOFA** $\ge 2$ **and** $\Delta$**SOFA** $\le 0$): Accounting for 60.2% of the study cohort, this group is the core subgroup of initial severe disease with good treatment response. Patients in this group had organ dysfunction at admission, belonging to the high-risk population defined by traditional static scores, but organ function remained stable or improved within 72 hours, with the IMV rate reduced to 30.3%. This indicates that the initial disease severity is not the only determinant of prognosis in CAP patients, and the stable disease trajectory after early treatment can effectively reduce the risk of IMV. The identification of this subgroup provides a dynamic reference for the clinical “evaluation of treatment response in severe patients”.
- **Group D (High-risk deteriorating: admission SOFA** $\ge 2$ **and** $\Delta$**SOFA** $> 0$): Accounting for 21.2% of the study cohort, this group is the extremely high-risk population among CAP patients. Patients in this group had both admission organ dysfunction and 72-hour deterioration, with an IMV rate as high as 81.3%, making them a high-risk population for IMV. The identification of this subgroup provides a clear target for the precise screening of high-risk populations at the 72-hour clinical node.

Third, the comparative analysis of static and dynamic prediction models showed that: the static model only included admission CURB-65, PSI and admission SOFA risk, with an AUC of 0.741; the dynamic model added the $\Delta$SOFA trajectory (*delta_risk*) on this basis, and the AUC was significantly increased to 0.850 ($\Delta$AUC = 0.109, $P<0.001$), with good calibration (Hosmer–Lemeshow $P=0.443$, Brier score 0.149) and significant reclassification improvement (NRI = 0.482, IDI = 0.192, both $P<0.001$). Multivariable logistic regression further quantified the risk gradient of the four ABCD subgroups: compared with Group A, the adjusted odds ratios of IMV for Groups B, C, and D were 12.60 (95% CI 3.32–56.94), 5.78 (95% CI 2.07–20.99), and 91.81 (95% CI 28.47–375.09), respectively (all $P<0.001$).

Fourth, decision curve analysis showed that within the clinically relevant threshold range (0–0.5) at the 72-hour node, the net benefit of the dynamic model was higher than that of the static model, with net benefit AUCs of 0.1287 (dynamic) and 0.1063 (static), respectively; the optimal risk threshold of the dynamic model was 0.37, with a sensitivity of 65.5% and a positive predictive value of 77.2%, which could identify high-risk patients more accurately and provide a quantitative reference for risk screening based on ABCD stratification at the 72-hour node.

### Comparison with existing literature

The findings of this study are consistent with previous research emphasizing the prognostic importance of dynamic changes in clinical status for community-acquired pneumonia (CAP). Building on this foundation, the study achieves one key expansion: constructing a clinically implementable ABCD stratification framework and verifying the incremental predictive value of $\Delta$SOFA through the dynamic model, which addresses the limitation that previous dynamic indicators “can only be statistically verified but are difficult to apply in clinical practice”.

Previous risk assessment tools for CAP (such as PSI, CURB-65) are all based on static admission data, which can achieve admission risk stratification but cannot capture the treatment response and disease evolution of patients within 72 hours, and this trajectory is the key factor determining the risk of IMV^8^. This study confirmed that $\Delta$SOFA, as a core indicator reflecting early disease evolution, can supplement significant incremental predictive value to static scores, which is consistent with the research conclusion of “dynamic variables improving predictive efficiency” in other infectious diseases. At the same time, different from previous studies focusing only on a single dynamic indicator, this study combined admission SOFA with $\Delta$SOFA to construct an ABCD dual-dimensional stratification framework, transforming dynamic indicators from “a single statistical quantity” into “four clinically identifiable risk populations”, which is more in line with conventional clinical diagnosis and treatment thinking.

To our knowledge, this is the first study to construct a 72-hour dynamic risk stratification framework for hospitalized CAP patients and complete a full-dimensional quantitative verification through a dynamic model. The ABCD stratification framework is intuitive and easy to operate, which can be achieved with only two SOFA scores. The dynamic model quantitatively verifies the incremental value of $\Delta$SOFA and provides statistical support for the ABCD stratification framework. The combination of the two transforms “dynamic risk assessment” from research conclusions into clinically operable tools, providing empirical support for the paradigm shift of CAP risk assessment from static admission to dynamic trajectory.

### Limitations and methodological considerations

When interpreting the results of this study, the following limitations should be fully considered, and all conclusions are only applicable to CAP patients who are hospitalized for more than 72 hours and can complete dynamic SOFA assessment.

***Study Design and Dataset Limitations*** This is a single-center retrospective analysis based on a single public dataset from a tertiary hospital in Colombia, which inherently limits the generalizability of our findings. Therefore, the results may not be directly applicable to CAP patients in other regions, healthcare systems, community hospitals, or populations with different demographic or clinical characteristics. Differences in IMV intubation thresholds, ward monitoring processes, and CAP treatment protocols among different institutions may affect the incidence of IMV and the predictive performance of the model. Multicenter external validation across diverse settings is urgently needed before the ABCD framework can be considered for widespread clinical use.

***Outcome Ascertainment*** Although the decision to initiate IMV was not governed by a rigid, study-specific protocol, it was made by a multidisciplinary team of specialists using standardized diagnostic and monitoring criteria from the original NACef study^14^. This approach enhances the clinical representativeness of our findings, as it reflects real-world decision-making rather than an artificial study rule. Nevertheless, unmeasured variability in intubation thresholds across different centers remains a potential limitation, underscoring the need for external validation.

***Handling of Missing Data*** Reasonable statistical imputation was performed for missing data of some baseline indicators in this study: missing admission PSI score (*admission_psi*) data of 4 patients were filled with the median; missing age data of 7 patients were imputed with the median; missing gender data of 1 patient were imputed with the mode.and missing baseline BMI data were imputed using multiple imputation by chained equations (MICE) with 5 imputations. The imputation model included age, sex, CURB-65, PSI, and admission SOFA to preserve the relationships among these variables. Convergence was assessed by inspecting trace plots of imputed values across iterations. The pooled estimates from the five imputed datasets were used for descriptive analyses (e.g., Table 1). Of note, BMI was not included as a predictor in any of the multivariable regression models; therefore, the handling of missing BMI data does not affect the primary predictive analyses.For core stratification and outcome indicators, to ensure data integrity, 187 patients (24.3% of the initial 768 patients) with missing admission or 72-hour SOFA scores and missing IMV outcome data were excluded from this study. A complete-case analysis was adopted to avoid imputation bias of core indicators, but selection bias may be introduced: the comparison of baseline characteristics between included and excluded patients showed that the admission SOFA scores of excluded patients were significantly lower, mostly due to discharge or death before 72 hours. Therefore, the study cohort only represents the subgroup of CAP patients who have sufficient hospital stay to complete 72-hour dynamic assessment, and the study results are not applicable to CAP patients who are discharged, die within 72 hours after admission.

***Limitation of the 72-hour Reassessment Time Point*** The 72-hour time point was chosen as the SOFA reassessment window because it is a critical clinical node for evaluating the initial treatment response of CAP and is consistent with the time frame for defining CAP treatment failure. However, this design has clear time limitations and research gaps: the ABCD stratification and dynamic model of this study only have incremental value for IMV prediction at the 72-hour node, and are not applicable to IMV prediction at any time point within 72 hours after admission; the predictive value of the model after 72 hours has not been verified and should be extrapolated with caution. Meanwhile, this study only used the final value of $\Delta$SOFA at the 72-hour node for analysis, and did not conduct quantitative exploration on the progressive change process of SOFA score within 72 hours. The dynamic change of SOFA is a continuous clinical course performance, and its phased changes within 72 hours (especially the continuous trend of patients with high admission SOFA) may have important IMV early warning value. This study did not clarify its specific early warning threshold and efficacy, which is an important research gap.

***Definition Limitation of admission SOFA*** In this study, “admission SOFA” refers to the score calculated within 24 hours of admission, not the pre-morbid admission organ function status. $\Delta$SOFA only reflects the change in organ function from admission to 72 hours, not the absolute level of organ function. This definition has been clarified in the methodology section, and this limitation should be noted when interpreting.

***Unmeasured Confounding Factors*** Consistent with all observational studies, residual confounding bias caused by unmeasured variables may still exist in this study, such as microbiological test results, patient frailty status, advance directives, timing of antibiotic administration, and oxygen therapy strategies. This study tried to minimize bias by adjusting for key clinical confounding factors, but the possibility of residual confounding cannot be completely excluded.

***Sample Size Limitations in Subgroup Analyses*** The significant interaction between COPD and ABCD grouping ($P=0.0395$) warrants careful interpretation. While this may suggest that COPD modifies the predictive effect of $\Delta$SOFA trajectory, the extremely small sample sizes in COPD subgroups (e.g., only 2 patients in Group A and B with COPD) limit the reliability of this finding. Future studies with larger COPD cohorts are needed to validate whether the ABCD framework performs differently in this population.

### Clinical implications and 72-hour risk assessment pathway

Despite the above limitations, the core value of this study is to provide a set of risk assessment tools based on dynamic trajectory for the 72-hour node of hospitalized CAP patients. Its clinical significance is not to directly guide diagnosis and treatment intervention, but to supplement incremental risk stratification information for routine 72-hour disease reassessment and provide evidence-based reference for clinicians’ decision-making.

Based on the findings of this single-center study, we propose a practical, non-interventional 72-hour risk assessment pathway,which is only for clinical reference, does not replace clinical judgment, and is strictly limited to IMV risk stratification at the 72-hour node:

1. **Admission stage (0–24 hours):** Calculate PSI, CURB-65, and admission SOFA scores according to clinical routine, complete admission risk triage, and carry out standard initial CAP treatment; for high-risk patients with admission SOFA $\ge$ 2, SOFA can be included in the subsequent routine monitoring list to prepare for the calculation of $\Delta$SOFA and disease assessment at the 72-hour node.
2. **72-hour node reassessment (±2 hours):** On the basis of routine disease assessment, supplement the detection of 72-hour SOFA and calculate $\Delta$SOFA, complete dynamic risk assessment using ABCD stratification, and provide incremental IMV risk information:
  **Group A (Low-risk stable):** With an IMV rate of 5.8%, it indicates a low risk at the 72-hour node. The intensity of routine monitoring and treatment can be maintained to avoid unnecessary consumption of medical resources.
  **Group B (Low-risk deteriorating):** With an IMV rate of 33.3%, it is a moderate-risk population. It is recommended to increase the frequency of organ function monitoring, and the treatment response should be evaluated by the attending physician in combination with clinical practice.
  **Group C (High-risk stable):** With an IMV rate of 30.3%, it indicates that high-risk patients have stable conditions after early treatment. The existing treatment and monitoring intensity can be maintained, and there is no need to blindly upgrade intervention measures.
  **Group D (High-risk deteriorating):** With an IMV rate of 81.3%, it is an extremely high-risk population. It is recommended to conduct key comprehensive clinical evaluation, strengthen organ function monitoring, and optimize treatment plans.
3. **After 72 hours:** The ABCD stratification result is only a risk reference at the 72-hour node. Clinical dynamic monitoring of disease changes should still be carried out according to routine; if the patient’s clinical status fluctuates significantly, the SOFA score can be repeated and re-stratified, but its predictive value has not been verified and should be judged in combination with clinical practice.

In summary, the core of this pathway is to supplement a simple and operable dynamic risk stratification method in routine 72-hour reassessment to provide incremental IMV risk information, which is consistent with the core finding of this study that “72-hour $\Delta$SOFA has incremental predictive value”.

### Future directions

Based on the core achievements and limitations of this study, the following targeted research directions are proposed, among which exploring the early warning value of progressive changes in SOFA score is the core direction:

1. **Core direction: Explore the early warning value of progressive changes in SOFA score:** Aiming at the key gap of this study, conduct prospective studies to quantitatively evaluate the continuous change law of SOFA score in CAP patients within 72 hours after admission, focus on high-risk patients with admission SOFA $\ge$ 2, clarify the early warning threshold and time node of phased increase in SOFA score, and explore the early warning efficacy of continuous change trend of SOFA within 72 hours on IMV; at the same time, verify whether the stabilization or decrease of SOFA score in patients with high admission SOFA within 72 hours can be used as a quantitative reference signal for effective treatment, extending dynamic risk assessment from “72-hour node assessment” to “continuous whole-process early warning within 72 hours”, and further improving the timeliness of CAP risk assessment.
2. **Multicenter external validation:** Conduct prospective multicenter studies in different healthcare systems, regions, and hospital types (tertiary hospitals, community hospitals) to verify the generalizability of the ABCD stratification framework and dynamic model, and evaluate the impact of local clinical practice patterns (such as IMV intubation indicators) on the predictive performance of the model, so as to provide more extensive empirical support for its clinical application.
3. **Development of early prediction tools within 72 hours:** For CAP patients who require IMV within 72 hours and are not covered by this study, construct an IMV prediction model based on admission data or early clinical indicators, which complements the 72-hour dynamic stratification framework of this study to form a phased risk assessment system for CAP patients from admission to 72 hours.
4. **Expanding the prediction outcomes of the stratification framework:** The ABCD stratification framework of this study is only for IMV prediction. In the future, its predictive value for other adverse outcomes of CAP patients (such as in-hospital mortality, prolonged hospital stay) can be explored to improve the clinical application breadth of the framework.
5. **Integrating more accessible dynamic indicators:** This study only included SOFA score as a dynamic indicator. In the future, other clinically accessible dynamic indicators (such as respiratory rate, oxygenation index, peripheral blood inflammatory indicators, etc.) can be integrated into the stratification framework to further optimize its predictive efficiency while maintaining the clinical operability of the framework.
6. **Real-world application research:** After completing multicenter external validation and the early warning study on progressive changes of SOFA score, conduct small-scale real-world application research to evaluate the operability and acceptability of the ABCD stratification framework in routine clinical diagnosis and treatment, and provide practical basis for its subsequent clinical transformation.

### Conclusions

In summary, this single-center study demonstrates that, in CAP patients hospitalized for $\ge$72 hours, he ABCD dynamic stratification framework based on admission SOFA and 72-hour $\Delta$SOFA can significantly improve IMV risk prediction within this cohort. The dynamic model quantified the incremental value of $\Delta$ SOFA and supports the ABCD framework, suggesting that the 72-hour dynamic trajectory of organ function is an independent predictor of IMV in CAP patients. These findings highlight the potential of incorporating dynamic changes into risk assessment, complementing traditional static scores. However, multicenter external validation is required to confirm these results and assess generalizability.

The four ABCD subgroups have independent clinical connotations: Group A is a low-risk reference population, Group B is an early warning population for mild disease progression, Group C is a reference population for effective treatment of severe disease, and Group D is an extremely high-risk screening population, which jointly constitute a complete 72-hour dynamic risk map and can provide clinicians with intuitive and operable IMV risk stratification information in routine reassessment.

The clinical value of this study is to provide a set of evidence-based dynamic risk assessment tools for routine 72-hour disease reassessment of CAP patients, promoting the transformation of CAP risk assessment from a single static admission to a comprehensive assessment of admission + dynamic trajectory. This study has limitations such as single-center retrospective design, focus only on the 72-hour node, and failure to explore the early warning value of progressive changes in SOFA score, and the generalizability of the study results should be treated with caution. In the future, the early warning value of progressive changes in SOFA score should be taken as the core research direction, combined with multicenter external validation, development of early prediction models within 72 hours and other studies, to further improve the dynamic risk stratification system, make it gradually transform into a risk assessment tool that can be routinely applied in clinical practice, and ultimately provide reference for the individualized diagnosis and treatment of CAP patients.

## Supplementary Information

Supplementary Information 1.

Supplementary Information 2.

Supplementary Information 3.

## Data Availability

The dataset analyzed during the current study is available from the PhysioNet repository under accession number: https://physionet.org/content/nacef/2.0.1/. Access to the data is restricted and requires completion of the PhysioNet credentialing process, which includes signing a Data Use Agreement and completing the CITI training on human subjects research.

## Abbreviations

ABCD: Stratification integrating admission SOFA and $\Delta$ SOFA (Groups A–D)
aOR: Adjusted Odds Ratio
AUC: Area Under the Receiver Operating Characteristic Curve
CAP: Community-Acquired Pneumonia
CI: Confidence Interval
CITI: Collaborative Institutional Training Initiative
COPD: Chronic Obstructive Pulmonary Disease
CURB-65: Confusion, Urea, Respiratory rate, Blood pressure, age $\ge$65 years
DCA: Decision Curve Analysis
HIPAA: Health Insurance Portability and Accountability Act
ICMJE: International Committee of Medical Journal Editors
ICU: Intensive Care Unit
IDI: Integrated Discrimination Improvement
IRB: Institutional Review Board
IQR: Interquartile Range
MIT-LCP: Massachusetts Institute of Technology Laboratory for Computational Physiology
MV: Mechanical Ventilation
NACef: Community-Acquired Pneumonia, Endotypes, and Phenotypes
NPV: Negative Predictive Value
NRI: Net Reclassification Index
OR: Odds Ratio
PPV: Positive Predictive Value
PSI: Pneumonia Severity Index
ROC: Receiver Operating Characteristic
SD: Standard Deviation
SOFA: Sequential Organ Failure Assessment
$\Delta$ SOFA: Change in SOFA Score
