# Supplementary material for: 72-hour SOFA changes and risk stratification for invasive mechanical ventilation in patients with community-acquired Pneumonia
Source: Sci Rep. 2026 Mar 17;16:13815. doi: 10.1038/s41598-026-44586-2 (PMC13128861; doi:10.1038/s41598-026-44586-2)
Supplement: Supplementary file 1 — Supplementary Information 1. [file 41598_2026_44586_MOESM1_ESM.pdf]

5 Supplementary Material

Table S1: Model Performance in Training Set and 10-Fold Cross-Validation

Metric	Training Set	Cross-Validation (Mean ± SD)	Δ (Train − CV)
AUC	0.851	0.847 ± 0.054	0.004
Sensitivity	0.815	0.802 ± 0.107	0.013
Specificity	0.766	0.761 ± 0.055	0.005
Brier Score	0.149	0.151 ± 0.026	–0.002

Note: The minimal differences between training and cross-validated performance (AUC $\Delta = 0.004$) and the relatively small standard deviations indicate no substantial overfitting and good generalizability of the model.

Table S2: Subgroup analysis of IMV rates across ABCD risk groups

Subgroup	Category	Group A	Group B	Group C	Group D	P for interaction
Overall	Overall	5.8% (4/69)	33.3% (13/39)	30.3% (106/350)	81.3% (100/123)	–
Gender	Female	0% (0/27)	30% (3/10)	23.7% (32/135)	79.5% (31/39)	0.339
	Male	9.5% (4/42)	34.5% (10/29)	34.1% (73/214)	82.1% (69/84)	
Age	< 65	6% (3/50)	25% (7/28)	29.3% (61/208)	84% (63/75)	0.267
	≥ 65	5.3% (1/19)	54.5% (6/11)	31.7% (45/142)	77.1% (37/48)	
COPD	No	6% (4/67)	29.7% (11/37)	30.3% (94/310)	84.3% (91/108)	0.0395
	Yes	0% (0/2)	100% (2/2)	27.3% (12/44)	60% (9/15)	

Note: Data are presented as percentage (number of events/total number). P for interaction was calculated using logistic regression with likelihood ratio test. The significant interaction for COPD (P=0.0395) may be influenced by small sample sizes in subgroups (e.g., Group A and B with COPD had only 2 patients each), and should be interpreted with caution.

Table S3: Distribution of key characteristics across 10-fold cross-validation validation sets

**Fold**	**N**	**Age median (IQR)**	**Male (%)**	**admission SOFA ≥ 2 (%)**	**ΔSOFA >0 (%)**	**IMV (%)**	**COPD (%)**
1	58	58 (47.2–64.8)	67.2	84.5	27.6	41.4	13.8
2	58	64.5 (56.2–72.8)	59.6	84.5	36.2	53.4	10.3
3	59	60.5 (49.8–75)	62.7	86.4	28.8	40.7	11.9
4	58	56 (50–66)	72.4	75.9	20.7	29.3	8.6
5	58	54 (41–65)	65.5	70.7	25.9	44.8	5.2
6	58	59.5 (50–71)	48.3	77.6	27.6	32.8	12.1
7	58	59.5 (49.2–68)	58.6	77.6	31.0	32.8	6.9
8	58	67 (50.2–73)	69.0	89.7	32.8	44.8	17.2
9	58	61 (50–68.5)	70.7	86.2	19.0	31.0	15.5
10	58	61 (52.2–72.8)	62.1	81.0	29.3	32.8	6.9
Overall (range)	58–59	60 (54–67)	63.6 (48.3–72.4)	81.4 (70.7–89.7)	27.9 (19.0–36.2)	38.4 (29.3–53.4)	10.8 (5.2–17.2)

Note:Data are presented as median (interquartile range) for age, and percentage (range across folds) for categorical variables. The minimal variation across folds indicates balanced splits in the 10-fold cross-validation.

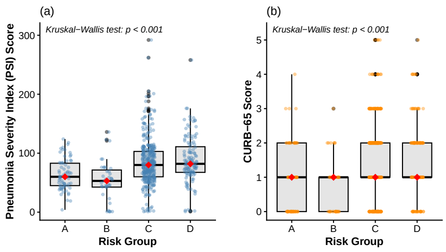

Figure S1: Distribution of pneumonia severity scores across four risk groups. (a) Pneumonia Severity Index (PSI) score; (b) CURB-65 score. Between-group differences were evaluated using the Kruskal-Wallis test (all $P < 0.001$).

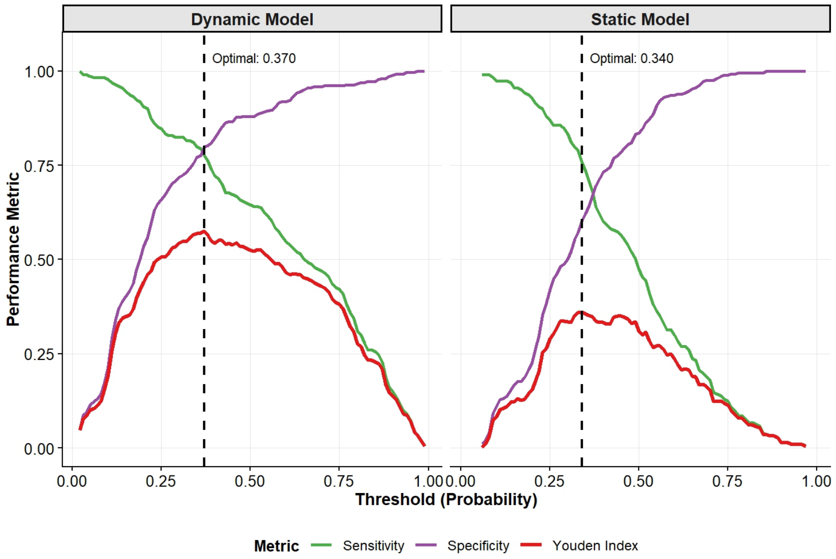

Figure S2: Performance metrics across probability thresholds for the two prediction models. Left panel: Model with Δ SOFA score; Right panel: Model without Δ SOFA score. The green, purple, and red curves represent sensitivity, specificity, and Youden index, respectively. The vertical dashed line indicates the optimal probability threshold determined by the maximum Youden index.
