# Supplementary figures and images for: 72-hour SOFA changes and risk stratification for invasive mechanical ventilation in patients with community-acquired Pneumonia
Source: Sci Rep. 2026 Mar 17;16:13815. doi: 10.1038/s41598-026-44586-2 (PMC13128861; doi:10.1038/s41598-026-44586-2)

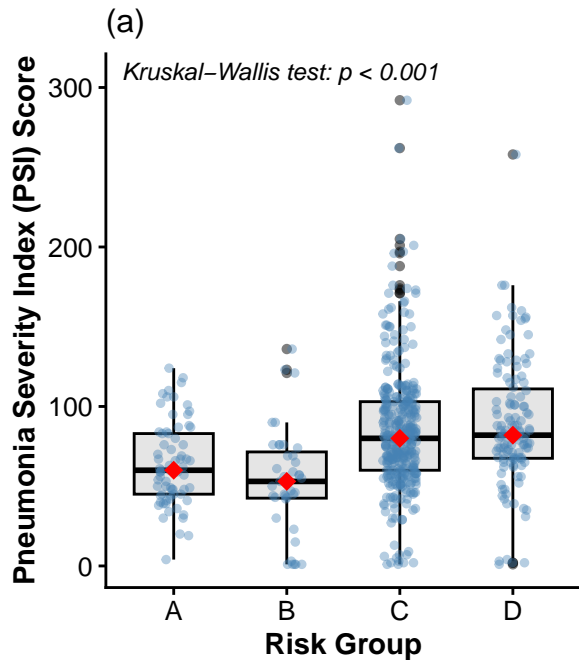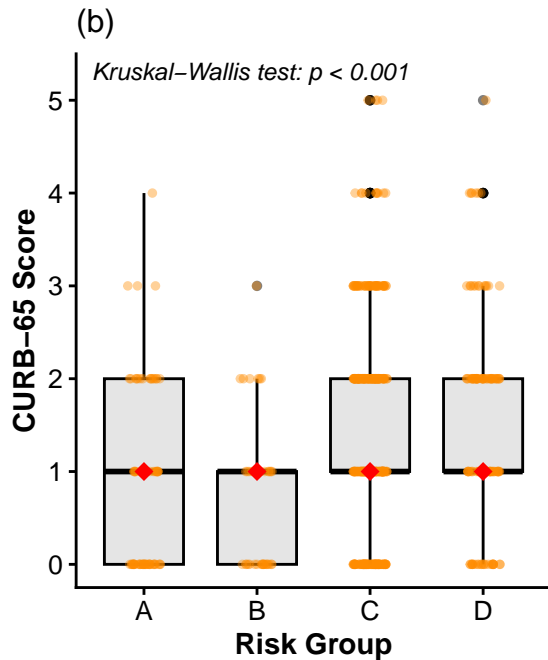

Supplement: Supplementary file 2 — Supplementary Information 2. [file 41598_2026_44586_MOESM2_ESM.pdf]

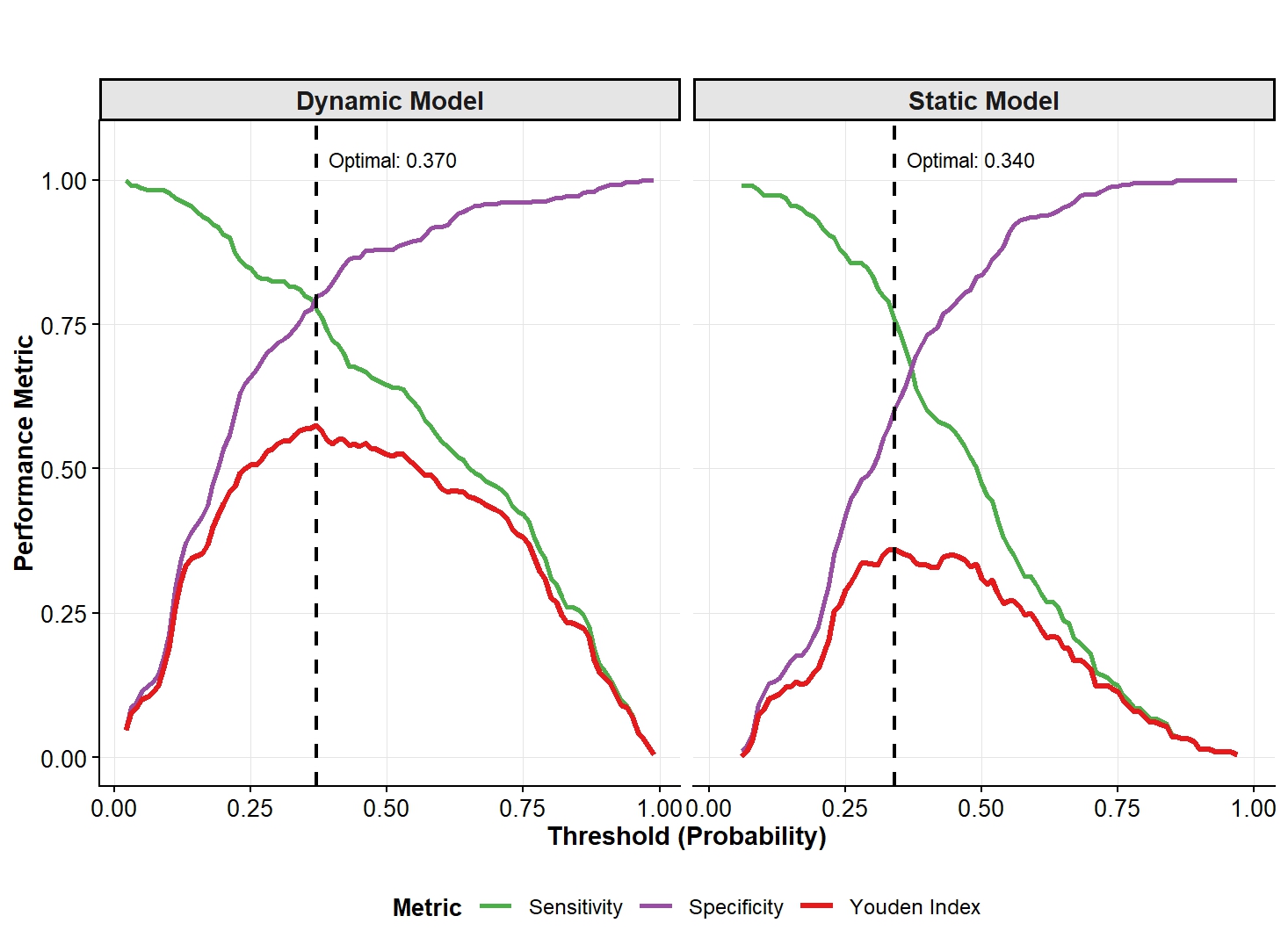

Supplement: Supplementary file 3 — Supplementary Information 3. [file 41598_2026_44586_MOESM3_ESM.jpeg]
